# Resignation of Professor Godman

**Published:** 1828-08

**Authors:** 


5. *Resignation of Dr. Godman.*—We regret to learn, that the health of our ingenious and estimable friend, professor Godman, of Rutgers' Medical College, New-York, has declined so much, as to make a resignation necessary. On receiving his communication, the President and Faculty resolved, that his ill health was deeply to be regretted, not only as a severe loss to the high standing of the Institution, but more especially for the lively interest they took in the personal welfare of one who had endeared himself to them, by his social virtues, no less than by his intellectual and professional endowments. Dr. Godman's diseases are, in a great degree, the result of an application which has raised him into a distinction which few men of his age have attained in this country. His successor has not yet we believe been appointed.

